# Sindbis Virus Platform Provides an Oncolytic-Virus-Mediated and Immunotherapeutic Strategy to Overcome the Challenging Microenvironment of Pancreatic Cancer

**DOI:** 10.3390/ph18050725

**Published:** 2025-05-15

**Authors:** Silvana Opp, Christine Pampeno, Alicia Hurtado, Daniel Meruelo

**Affiliations:** 1 Department of Pathology, NYU Grossman School of Medicine, New York University, New York, NY 10016, USA; silvana.opp@biontech.us (S.O.); christine.pampeno@nyulangone.org (C.P.); alicia.hurtadomartinez@nyulangone.org (A.H.); 2 BioNTech Ltd., Cambridge, MA 02139, USA

**Keywords:** Sindbis virus, oncolytic virus, immunotherapy, pancreatic cancer, IL12, OX40

## Abstract

**Background/Objectives:** Our laboratory has been developing a Sindbis viral (SV) vector platform for treatments of several types of cancers. In this study, we assess treatment efficacy for metastatic and immunosuppressive pancreatic cancer. **Methods:** Orthotopic mouse models were generated by injection of tumor cells into the pancreatic parenchyma. Sindbis vectors were inoculated intraperitoneally. Imaging of tumors was performed by either MRI or in vivo imaging using luciferase. Flow cytometry, multi-immunofluorescence and elispot analysis were performed for certain tumors. **Results:** SV can infect and reduce pancreatic tumors in three mouse model systems: a model bearing human pancreatic tumors, a highly metastatic model, and a model that reflects the highly immunosuppressive, desmoplastic microenvironment common to human pancreatic cancer. **Conclusions:** Combination of SV vector expressing IL12 with an immune co-stimulatory agent, anti-OX40, can reduce tumors, facilitate an influx of immune response cells into the tumor microenvironment, and prevent tumors in mice rechallenged with tumor cells promising an effective treatment for pancreatic cancer.

## 1. Introduction

A Sindbis viral (SV) vector expressing an immunomodulatory molecule, agonistic OX40 antibody (αOX40), combined with the cytokine IL-12, has shown strong efficacy in the treatment of highly malignant ovarian cancer preclinical models [1,2]. The success of these studies prompted evaluation of this vector for the treatment of pancreatic cancer, another lethal malignancy with poor prognosis.

Pancreatic cancer is typically diagnosed at a late stage when symptoms become apparent, and metastasis most likely occurs. Pancreatic ductal adenocarcinoma (PDAC), the most common type of pancreatic cancer (95%), has a five-year survival rate of only 13% [3]. Despite progress using chemotherapeutic agents, such as FOLFIRINOX and gemcitabine plus albumin-bound paclitaxel [4], <30% of patients respond to either regimen, and median progression-free and overall survival remain under 6 and 12 months, respectively [5].

A major challenge in PDAC treatment involves an immunosuppressive tumor microenvironment (TME) and a dense extracellular matrix known as desmoplasia [6,7,8,9]. The desmoplastic TME elevates interstitial fluid pressure that limits small-molecule perfusion and contributes to the restriction of nutrients and oxygen due to metabolic deregulation of tumor cells. Infiltrating T cells, requiring high energy demands, become dysfunctional in this environment [10,11].

Our studies involving multiple mouse tumor models indicate that combining an SV vector with agonistic antibodies to OX40 (αOX40) can confer complete remission and protection from recurrence by driving activated T cells selectively into the TME [1,12,13]. OX40 (CD134), a member of the tumor necrosis factor (TNFR) superfamily, is a co-stimulatory receptor that is expressed on activated T cells. Interaction between OX40 and its ligand, OX40L, promotes the clonal expansion, differentiation, and survival of CD4 Th1 helper cells, which produce IFNγ and IL-2 cytokines [14,15,16,17] that sustain the survival of primed CD8 T cells [18,19]. The co-expression of OX40 with ICOS on follicular T helper cells (Tfh) facilitates the differentiation of antibody-producing B cells and long-lived plasma cells from germinal center B cells [20]. In addition, OX40 signaling represses regulatory T cells (Treg) by downregulating the expression of Foxp3 [21].

The observation that IL-12 increases the presence of OX40 on the surface of CD4 T cells initiated the study of a combined anti-tumor capacity [13,22]. IL-12 activates T cells, stimulates the production of IFNγ and increases the expression of OX40 on effector CD4 T cells [22]. The combination of SV.IL-12 with an agonistic antibody to OX40 has been shown to exhibit strong therapeutic efficacy in CT26, colon, and MyC-CaP, prostate carcinoma, models [13]. SV expressing IL-12 combined with agonistic OX40 antibody changes the transcriptome and metabolic program of T cells, developing highly activated, terminally differentiated effector T cells, with enhanced tumor infiltration capacity that can overcome the repressive TME [1,12,13]. Intra-tumoral T cell immune responses showed increased granzyme B levels. Anti-tumor activity was observed by a decrease in Ki-67 proliferation marker in tumor cells. Early studies had shown that SV could significantly reduce subcutaneous tumors in a C.B-17-SCID mouse model bearing human pancreatic CFPAC cells [23] and in a syngeneic Pan02 C57BL/6 model [24]. The Pan02 cell line was also used to show that protein kinase R senses SV infection activating translational arrest, cellular stress, and apoptosis [25].

We have developed a Sindbis viral (SV) vector platform for the treatment of multiple types of tumors [1,12,13,24,26,27]. Sindbis is a positive single-strand RNA oncolytic alphavirus that can infect and specifically replicate in tumor cells, eliciting cell death and stimulating anti-tumor immune responses. The utility of alphaviruses as vector systems and oncolytic-virus-mediated therapy has been reviewed [28,29,30]. Reviews of their structure, expression, replication, and evolution have also been presented [31,32,33].

In this study, we demonstrate that SV vectors can effectively treat PDAC in three different preclinical model systems: human pancreatic cancer, a metastatic cancer model, and a model with an immunosuppressive TME.

## 2. Results

### 2.1. SV Vectors Can Generate Regression of Human Pancreatic Tumors in an SCID Mouse Model

**Figure 1:**
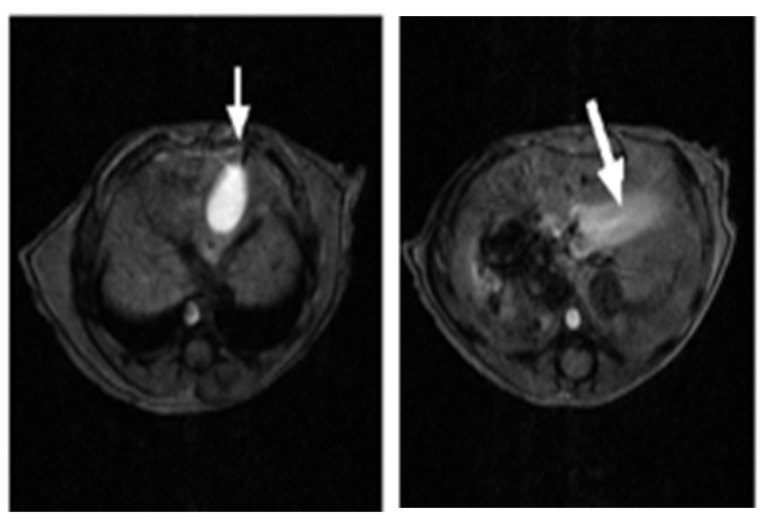
MRI MnDPDP images of control mice. (**Right**) T1-weighted images of upper abdomen of control animal at 7T. The gallbladder (arrow) appears bright. (**Left**) At a slightly lower level in the control animal, the pancreas (arrow) is brightly enhanced as compared to other intra-abdominal viscera.

To assess the ability of SV to infect and treat human pancreatic tumors, 2.5 × 10^6^ CFPAC-1 cells were injected directly into the pancreatic parenchyma of SCID mice following a modified protocol from Tomioka et al. [34]. Magnetic resonance imaging (MRI) was used to facilitate monitoring of tumor growth and therapy in these mice. At 21 days post-surgical implantation, animals were examined 30 minutes after IV injection of an MRI contrast medium, MnDPDP [manganese (II) N,N′-dipyridoxylethylene-diamine-N,N′-diacetate-5,5′-bis (phosphate)] (0.5 µmol/kg). Figure 1 shows that in an untreated control animal, contrast accumulated in the gallbladder as anticipated (right) and the pancreas was visualized as a bright band of signal in the expected location in the upper abdomen (left).

**Figure 2:**
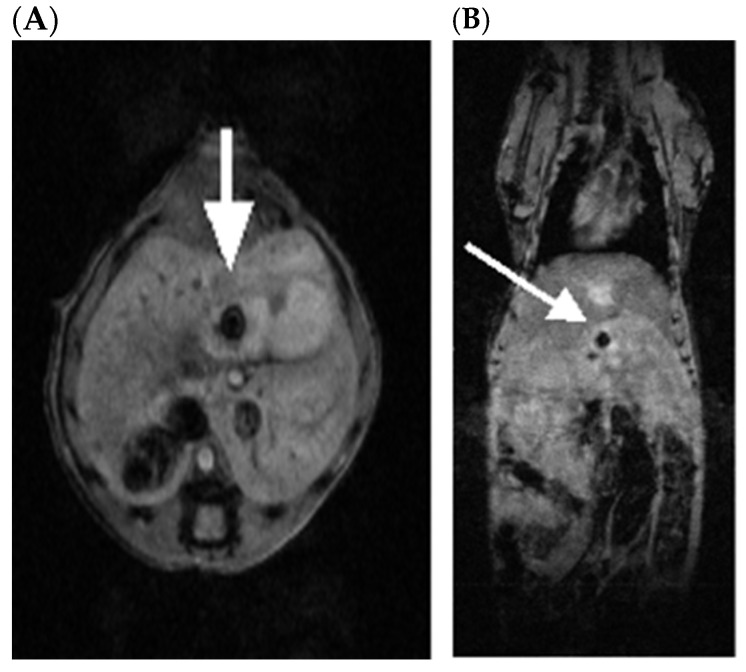
(**A**) Axial and (**B**) coronal images reveal low signal focus (arrows) in the pancreatic neck suspicious for tumor on day 21 after tumor cell inoculation. Imaging was performed using a gradient recalled echo (GRE) T1-weighted sequence. Following pilot scans to check alignment, T1-weighted GRE images were obtained in both the axial and coronal planes. Sequence parameters were as follows: TR 350 ms; TE 6 ms; NAV 8; Matrix 512 × 256 × 13 (coronal), 256 × 256 × 17 (axial); FOV 50 × 25 mm (coronal), 25 × 25 mm (axial); FA 65 deg. Spatial resolution was 98 × 98 × 750 µm.

**Figure 3:**
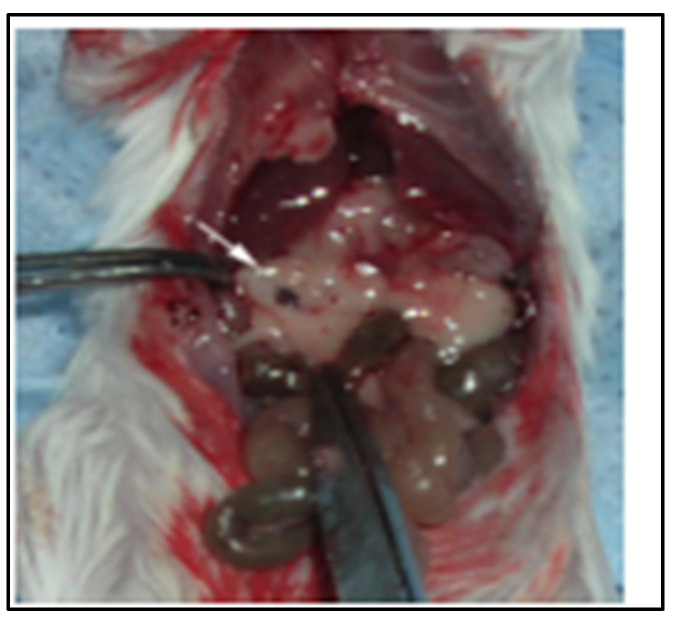
Photo of same mouse as in Figure 2. A nodular excrescence is seen in the pancreatic neck at the site corresponding to the MRI image (denoted by white arrow).

**Figure 4:**
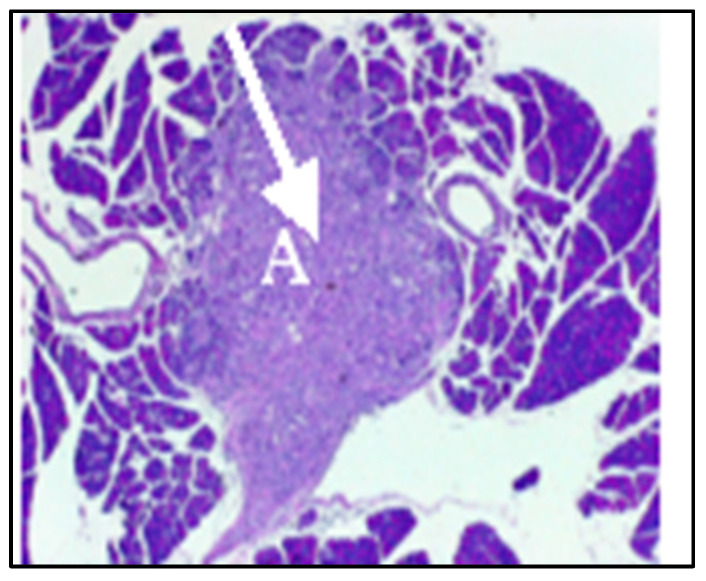
Histopathology of pancreatic tumor. Low-power view of pancreas of mouse in Figure 2. Discrete focus of tumor with features of adenocarcinoma (A).

**Figure 5:**
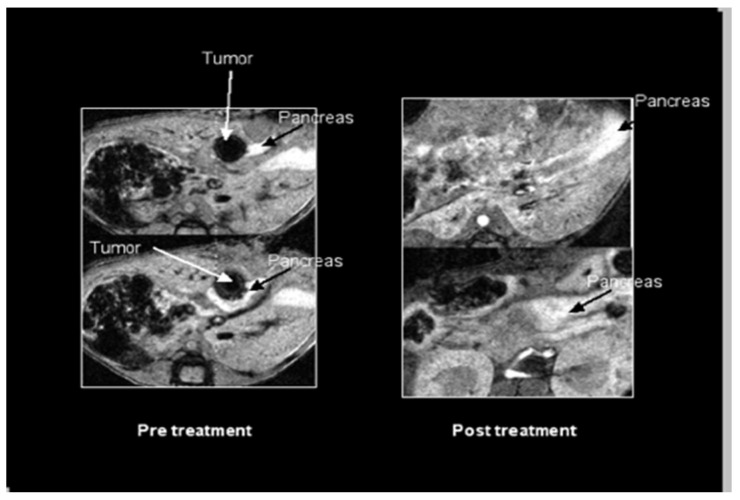
Administration of SV vectors to SCID mice with human pancreatic tumor cells growing orthotopically in the pancreas leads to eradication of the pancreatic tumor cells.

In tumor-bearing mice, each tumor appeared as a dark focus in the pancreatic neck (Figure 2). Following the initial imaging experiment, one animal was sacrificed and the pancreas was inspected for gross tumors and evaluated by histopathology with hematoxylin and eosin staining. On gross inspection, a nodular mass could be recognized in this location (Figure 3). Histologic evaluation confirmed this to be adenocarcinoma (Figure 4). Having established the model, we tested a second set of animals to determine whether SV vectors could prove therapeutic. As before, the pancreas was surgically exposed and CFPAC-1 cells were injected into each of five SCID mice. Animals were imaged 21 days after surgical implantation. Tumors were detected in all the injected mice. Following imaging, mice were treated daily for 2 weeks with SV vectors (~10^8^ TU per day/5 days per week) to determine whether tumor regression could be induced in the pancreas and whether such regression was detectable on re-imaging. In this model, treatment with SV vectors caused complete tumor regression of orthotopically implanted human pancreatic cancer cells growing in the pancreas of SCID mice (Figure 5).

### 2.2. SV.IL-12 Can Reduce Metastasis in a Pan02 Syngeneic Mouse Model

To address the impact of our therapy on metastasis, we used the Pan02 cell line derived from a pancreatic tumor that developed in a C57BL/6 mouse following implantation of 3-methylcholanthrene-soaked cotton threads into pancreas tissue [35]. Pan02 tumors have intrinsically high resistance to a wide range of chemotherapeutic agents and significant metastatic burden [35,36]. At 5 weeks post-Pan02 tumor implantation, when metastases were observed to occur mice were treated daily, five times per week, for 4 weeks with 10^8^ TU of SV vector expressing IL-12 (SV.IL-12). A comparison of the anti-tumor response seen against Pan02 metastases is shown on Table 1. SV.IL-12 was found to significantly reduce metastatic growth; however, complete remission was not observed. Previously, it has been shown that the IL-12 cytokine alone, i.e., without the vector, offers very little if any protection [13].

### 2.3. Effect of SV Vector on PDAC Models with Immunosuppressive TME

A limitation of the Pan02 model is that the orthotopic implants do not develop as pronounced a desmoplastic reaction as seen in human PDAC tumors [37,38]. KC and KPC models are more analogous to human PDACs. Histological analyses of implanted KC and KPC cell lines demonstrate similar levels of fibrosis and leukocyte infiltration to those that arise in genetically engineered mouse models of spontaneous PDAC but express far fewer neoantigens [37,38].

**Figure 6:**
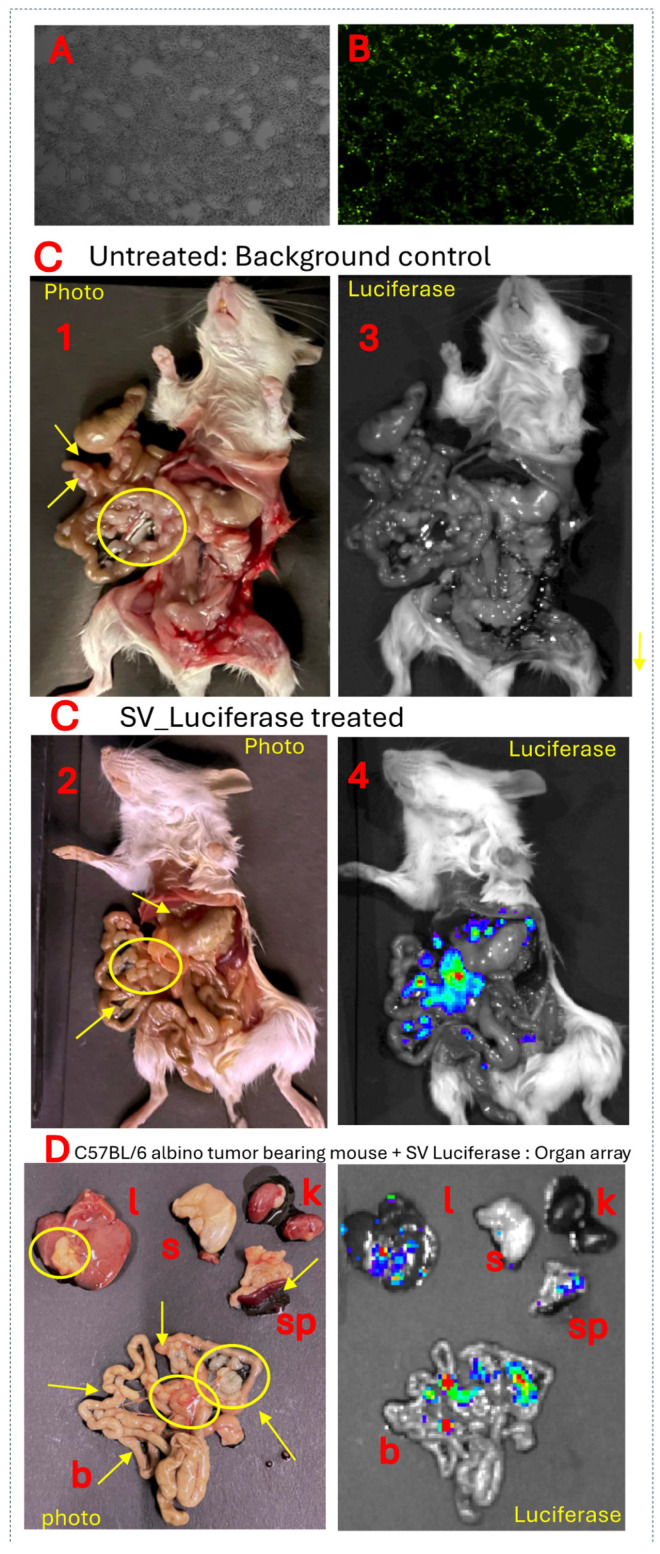
Infection and targeting in vitro and in vivo of pancreatic tumor cells by SV vectors. (**A**) Uninfected UN-KC-6141. (**B**) Cells infected with SV-GFP. (**C**) C57BL/6 albino UN-KC-6141 tumor-bearing mice. **Photos** (**1** and **2**). IVIS images of mouse not injected with SV. Luc (**3**) serves as a bioluminescence background control. Mouse treated with SV.Luc vector (**4**). (**D**) Organ array of SV-vector treated mouse in C showing specific tumor-targeting Sindbis vectors: (l) liver, (s) stomach, (k) kidneys, (sp) spleen and pancreas, (b) bowel. Arrows and circles indicate tumors.

**Figure 7:**
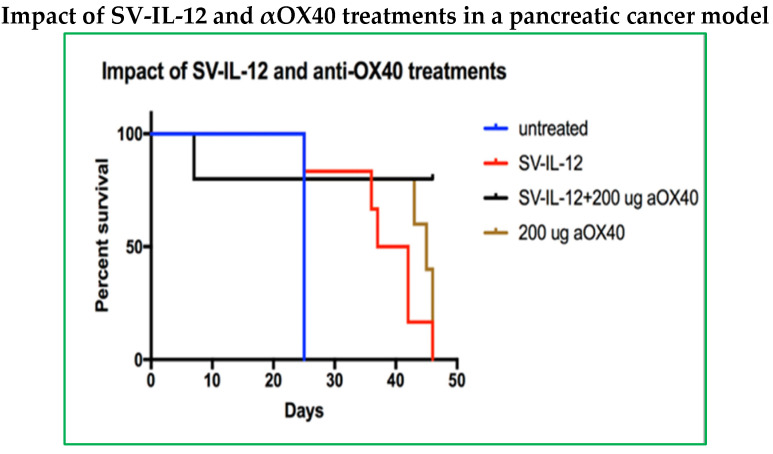
In the UN-KC-6141 pancreatic cancer model (C57BL/6a mice), SV vectors plus αOX40 markedly decrease tumor growth, leading to survival of most animals treated with the combination.

UN-KC-6141 cells were derived from a pancreatic tumor of a *KrasG12D;Pdx1-Cre* (KC) mouse engineered to express gene mutations commonly observed in pancreatic cancer patients. These mice spontaneously produce tumors with features of human PDAC [39]. Figure 6 shows that SV expressing firefly luciferase (SV.Luc) was observed by IVIS imaging to specifically infect C57BL/6 albino orthotopic UN-KC-6141 tumors. This model, therefore, provides a means to test SV combination immunotherapy for the treatment of the immunosuppressive TME and desmoplasia characterizing PDAC. The results indicate that the combination of SV with IL-12 and αOX40 increases the therapeutic potential (Figure 7). In this experiment, UN-KC-6141 cells expressing firefly luciferase were used for IVIS imaging to monitor tumor growth. Four days after tumor inoculation, groups of mice (*n* = 25) were treated for one month with the indicated SVs (10^6^ TU, 4 days/week) and OX40 antibody (200 µg), as indicated, 3 days per week. Control mice (*n* = 10) were untreated.

**Figure 8:**
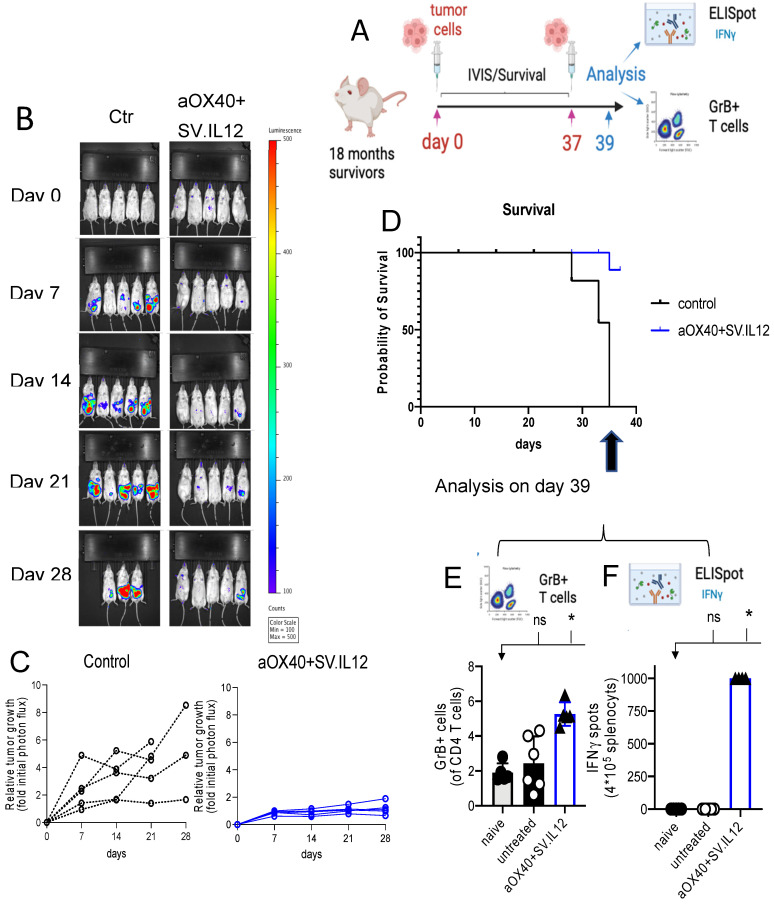
SV.IL12 + αOX40 protects from tumor recurrence in vivo. Eighteen months after SV.IL12 + αOX40 treatment, survivors were re-challenged with 5 × 10^5^ UN-KC-6141-Luc tumor cells. Untreated mice were used as control. A sketch of the experimental setup is shown in (**A**). Therapeutic efficacy of long-term protection was monitored by tumor luminescence, relative tumor growth (**B**,**C**), and survival (**D**). Noninvasive bioluminescent imaging was performed using the IVIS Spectrum imaging system (Caliper Life Science) at the indicated time points. To analyze immune-cell-mediated anti-tumor responses associated with long-term protection in the same re-challenged survivors, we reinjected them with UN-KC-6141 tumor cells on day 37 and excised spleens and stained a single-cell suspension for flow cytometry analysis on day 39. Naïve (mice not previously challenged with tumor or treated) and untreated mice (mice challenged with tumor cells on day 37) were used as controls. Shown is the percentage of granzyme B expression by CD4 T cells (**E**). Interferonc**γ** (IFN **γ**) enzyme-linked immunospot analysis of splenocytes from naïve, control, and survivor mice, as indicated (**F**). Bars represent means and each symbol represent an individual mouse. Statistical significance was determined with the Kruskal–Wallis test followed by the Dunn or Mann–Whitney test. Symbol, * indicates significance, ns is not significant.

Figure 8 demonstrates that SV.IL-12 vectors with αOX40 therapy confer long-lasting protection. A schematic of the experiment is shown in Figure 8A. UN-KC-6141-Luc tumor-bearing mice, surviving 18 months after treatment with SV.IL12 + αOX40, were re-challenged with 5 × 10^5^ UN-KC-6141-Luc tumor cells. Age-matched naïve mice received similar inoculation of tumor cells. Figure 8B,C show that by both photon flux and relative growth, animals previously cured by treatment with the SV.IL12 and αOX40 therapy are more protected than age-matched naïve mice. Survival of re-challenged mice was seen in 80% of those previously cured compared with naïve untreated mice (Figure 8D). To analyze immune-cell-mediated anti-tumor responses associated with long-term protection in the same re-challenged survivors, we reinjected them with UN-KC-6141 tumor cells on day 37, and then excised spleens and stained a single cell suspension for flow cytometry analysis on day 39. Naïve (mice not previously challenged with tumor or treated) and untreated mice (mice challenged with tumor cells on day 37) were used as controls. The results showed that the re-activation of granzyme B-expressing T cells (Figure 8E) and upregulation of IFNγ secretion (Figure 8F) helped to prevent tumor regrowth. No treatment with SV.IL12 + αOX40 was administered in this experiment. The observed protection results from memory T cell responses. Mechanistically, SV.IL-12 + αOX40 therapy achieves regression-free survival by driving activated T cells selectively and measurably into the cold, immunosuppressed tumor [12]. Contributing to this effect is that the combination of systemically administered SV vectors and αOX40 markedly changes the transcriptome signature and metabolic program of T cells, driving the development of highly activated, terminally differentiated, effector T cells. These reprogrammed T cells demonstrate enhanced tumor infiltration capacity, as well as anti-tumor activity throughout the body, overcoming the repressive TME [13,40].

**Figure 9:**
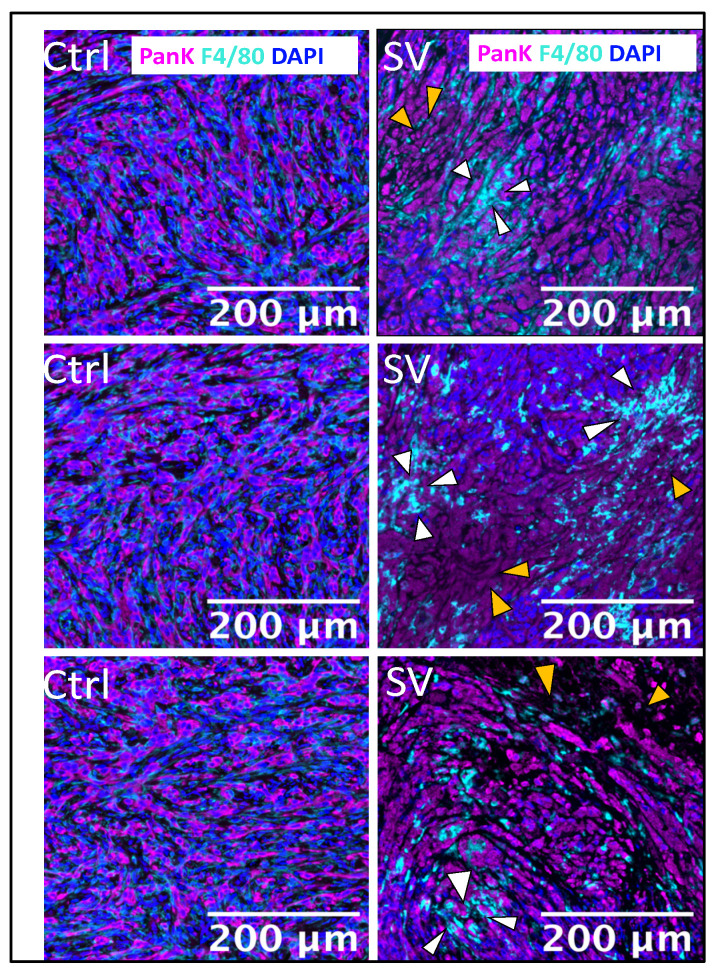
SV triggers tumor cell death in mice. Tumors were harvested after 2 weeks of treatment from untreated (control) and SV-treated mice. Tumors were stained by multiplex immunofluorescence. Three representative images are shown for one control mouse (**left panel**) and SV-treated (**right panel**) mice. Proteins of interest were stained and are indicated by color in each image: PanK (magenta) indicates tumor cells, F4/80 (Cyan) indicates macrophages, and DAPI nuclear staining appears in blue. White arrows indicate patches of recruited macrophages into areas of tumor cells (yellow arrows) undergoing cell death (indicated by PanK-positive cells and loss of DAPI) as a result of treatment.

SV tumor targeting and apoptosis can be similarly observed in the UN-KC-6141-pancreatic cancer model by use of multicolor immunohistochemistry. Staining with Pan cytokeratin (PanK) shows the presence of tumor cells that are killed by treatment with SV. Macrophage migration into the tumor TME induced by SV is revealed by staining with F4/80 antibody (Figure 9).

## 3. Discussion

Pancreatic cancer treatment requires targeting of primary and metastatic tumor cells as well as the desmoplasmic TME. Combinatorial treatment strategies that target both the TME and tumor cells of PDAC have been studied and multiple clinical trials are underway [9]. These include combining immune checkpoint blockade antibodies with drugs that target stromal elements or immunosuppressive myeloid cell inhibitors. Like OX40, another co-stimulatory TNFR, CD40, has been combined with chemotherapy. CD40 activates antigen-presenting cells (APCs) that can elicit an immune response to tumor antigens. When combined with chemotherapy, CD40 antibody agonists activate APCs to destroy tumor stroma and drive anti-tumor T cell responses in mouse models [41]. CD40 immunotherapy, in combination with gemcitabine and nab-paclitaxel chemotherapy, is currently in phase II clinical trials (ClinicalTrials.gov, NCT03214250) [42].

Oncolytic viruses (OVs) have been recognized as an immunotherapy that can overcome challenges presented by the TME. By selectively infecting and lysing tumor cells, OVs release tumor-associated antigens and danger-associated molecular patterns that trigger IFN type I responses, promoting antigen-presenting cell maturation that activates T cells. Preclinical and ongoing clinical trials have been described that employ OVs as monotherapy, with some expressing transgenes that target the TME or enhance the anti-tumor immune response. Combinations of OVs with immune checkpoint blockades and/or chemotherapy are also under investigation (reviewed in [43,44]).

Investigations related to our SV.IL12, αOX40 therapy have been performed. A long-term follow-up phase I clinical trial (2017–2024) of an adenovirus expressing IL12 has shown promising results for the treatment of metastatic cancer (ClinicalTrials.gov, NCT02555397) [45]. The adenovirus vector, inoculated intra-tumorally, was combined with chemotherapy in this study. Patients showed increased overall survival and elevated serum levels of immunostimulatory cytokines IL12 and IFNγ, and the chemokine CXCL10. The results of this study have encouraged upcoming phase II/III clinical trials.

Two TNFR family superfamily member co-stimulatory ligands, CD40L and 4-1BBL, have been encoded within an adenovirus, LOAd703, for intra-tumoral injection into PDAC tumors. LOAd703 treatment, combined with chemotherapy, was evaluated in a phase I clinical trial (ClinicalTrials.gov, NCT02705196) [46]. The results showed enhanced immune response and the study will continue with the addition of anti-PD1 inhibitor, atezolizumab.

In a preclinical study, OX40 ligand (OX40L) was inserted into the herpes simplex OV (HSV-1) and treatment of PDAC was evaluated in the KPC syngeneic mouse model [47]. The results support our data showing that OX40 stimulation suppresses tumor growth and prolongs survival (Figure 8). HSV-1 OV-mOX40L activated CD4 and CD8 T cells, as evidenced by increased IFNγ and granzyme b production, and increased migration of immune cells into the TME, similar to our observations with the UN-KC-6141 tumor model (Figure 9). In another study, the addition of αOX40 to an anti-PD1 checkpoint blockade was also shown to greatly augment the efficacy of treatment in the KPC PDAC model in the absence of OV therapy [48].

This report demonstrates that our SV platform promises to be an effective treatment for PDAC. SV was shown to reduce CFAC-1 human tumors in a xenotrophic SCID mouse model.

SV.IL12 also targeted and reduced tumors in a metastatic Pan02 syngeneic mouse model. Most notably, SV.IL12 combined with αOX40 was shown to infiltrate the desmoplasia of UN-KC-6141 pancreatic tumors resulting in tumor lysis, long-term protection against recurrence, and increased infiltration of macrophage cells.

The SV platform affords certain advantages over other oncolytic viruses: (1) Alphaviruses, like SV, are known to target lymph nodes and infect monocytes, macrophages, and dendritic cells, thus promoting the activation and priming of T cells [1,49,50,51,52]. Consequently, SV vectors can take advantage of the existing T cell repertoire, obviating prior knowledge of tumor-associated antigens (TAAs). (2) Sindbis virus is transmitted via mosquito bites to mammals [32]. As a blood-borne vector, SV can be administered via intravenous (i.v.) and intraperitoneal (i.p.) routes. The hematogenous delivery property of Sindbis virus enables it to reach tumor cells throughout the circulation, avoiding more complicated intra-tumoral injections [24]. (3) Studies show that repeated administration of vectors remains efficacious [53,54,55]. (4) SV has the safest profile among alphaviruses, with mostly asymptomatic infections in the wild [56,57]. SV is an RNA virus without replicative DNA intermediates that pose any risk of chromosomal integration or insertional mutagenesis [58]. To avoid even transient adverse effects, our vectors have been attenuated by splitting the SV genome and by removing the packaging signal from the genomic strand that encodes the structural genes [59,60].

In summary, we have shown in colon, prostate, and ovarian cancer models [1,13], and here in a pancreatic cancer model, that SV expressing IL12 in combination with stimulatory αOX40 can effectively reduce tumor burden, provide long-term protection, and stimulate anti-tumor immune responses. An SV has been generated that expresses both IL12 and αOX40.

## 4. Materials and Methods

### 4.1. Cell Lines

Human CFPAC-1 PDAC were obtained from the American Type Culture Collection (Manassas, VA, USA) (ATCC, CRL-1918); and cultured in DMEM (low-glucose modified, JRH Biosciences; Lenexa, KA, USA) supplemented with 10% FBS. Pan02 cells were obtained from the NCI–Frederick Cancer Research Facility (Frederich, MD, USA) and were maintained in DMEM supplemented with 10% FBS. The UN-KC-6141 cell line, a gift from the Surinder K. Batra laboratory (Univ. of Nebraska Medical Center, Omaha Nebraska, NE, USA), was cultured in DMEM medium supplemented with 10% FBS.

For IVIS imaging, a firefly luciferase (Luc)-expressing UN-KC-6141 cell line (UN-KC-6141-Luc) was generated by stable transfection of the pGL4.20_Fluc plasmid (Promega, Madison, WI, USA). All media were supplemented with 100 μg/mL of penicillin–streptomycin and 0.5 μg/mL of amphotericin B (Mediatech, Inc., Manassas, VA, USA). Cells were incubated at 37 °C, 5%CO_2_.

### 4.2. SV Production

The SV vectors used in these studies are propagation-defective and were produced as previously described [13,23,61]. Briefly, plasmids carrying the replicon or Sindbis helper RNAs were linearized with *PacI*, *NotI*, *or Xhol*, and in vitro transcribed using mMessage mMachine T7 or SP6 kits (InVitrogen, Thermo Fisher, Fairlawn, NJ, USA) according to protocol. RNA quality was assessed by gel electrophoresis and 18 µL of helper and replicon reactions was co-electroporated (25 µF, 1.2 kV) into 6 × 10^6^ BHK-21 cells. Electroporated cells were incubated in 10 cm dishes at 37 °C overnight. Cells were washed with PBS and 8 mL of Opti-MEM (minus FBS) (Thermo Fisher, Fairlawn, NJ, USA) supplemented with 100 mg/L CaCl_2_. After 24 h, supernatant was separated by centrifugation at 3000 rpm, for 15 min, and aliquoted for storage at −80 °C. Vectors were titrated in BHK cells, expressed as transducing units (TU) per ml. In SV.IL12, the IL-12 sequence is a synthetic construct of bioactive single-chain murine interleukin 12 mRNA [Genbank Sequence ID: AP411293.l]. SV.Luc expresses firefly luciferase excised from the pGL3 plasmid (Promega, Madison, WI, USA) [61].

### 4.3. Animal Experiments and Tumor Models

All experiments were in accordance with the Institutional Animal Care and Use Committee of New York University Langone Health [Approval Record: IA17-00796, 7/31/23]. Mice were used at 8 weeks of age. For imaging experiments, isoflurane was used as an anastatic. The CFPAC-1 human PDAC model was established by injection of 2.5 × 10^6^ cells into the pancreatic parenchyma of SCID mice (Jackson Labs, Bar Harbor, ME, USA) by a modified protocol from Tomioka et al. [34]. Groups of 5 mice were used for control and experimental treatments. Imaging was performed at the Animal Facility and the Preclinical Imaging Laboratory at NYU Langone Health as described in the text. The metastatic Pan02 model was established by orthotopic injection of 2.5 × 10^6^ cells into the pancreatic parenchyma of C57 BL/6 mice (Jackson Labs, Bar Harbor, ME, USA). Treatment with SV.IL12 (10^8^ TU) began 5 weeks after tumor cell inoculation; i.p., 5 times per week for 4 weeks. Groups of 5 mice were used for control and experimental treatments. The KPC orthotopic model was established by intra-pancreal injection of 5 × 10^5^ UN-KC-6141 cells into C57BL/6 albino mice, B6 (Cg)-Tyr-21/J, (Jackson Labs, Bar Harbor, ME, USA) to facilitate noninvasive bioluminescent imaging using the IVIS (in vivo imaging system) Spectrum Imaging System (Caliper Life Sciences, Hopkinton, MA, USA). Mice were randomized into untreated and treated groups for survival experiments. Tumor growth was quantified using the Living Image 3.0 software (Caliper Life Sciences, Hopkinton, MA, USA) as previously described [27]. Briefly, mice were injected with 150 mg/kg D-luciferin firefly and potassium salt (GoldBio, St. Louis, MO, USA), and luminescence was assessed 5 min later. Relative tumor growth was calculated individually for each animal by dividing the total body bioluminescence signal of a given day by the first one registered before treatment. As per the indicated treatment strategy, mice were injected i.p. with indicated doses of SV vectors and/or a commercial αOX40 antibody (dose—250 μg/mouse, BioXcell, Lebanon, NH, USA BE0031).

### 4.4. Histochemistry and Multiplex Immunofluorescence (MIF)

Tumors of mice were collected, fixed in 4% paraformaldehyde (PFA) for 2 days, embedded in paraffin, sectioned, and hematoxylin and eosin-stained. For MIF staining and imaging, 5 μm paraffin sections were stained with an Akoya Biosciences Opal multiplex automation kit (Akoya Biosci., Marlborough, MA, USA; 01752) on a Leica BondRX autostainer (Leica Biosystems, Lincolnshire, IL, USA), according to the manufacturer’s instructions as previously described [1]. Pancytokeratin (C-11) and macrophage F4/80 (EPR26545-166) were obtained from Abcam, Cambridge, UK.

### 4.5. Flow Cytometry Analysis of Granzyme B on CD4 T Cells and Elispot Analysis of IFNγ

For flow cytometry and elispot analysis, spleens were mashed though a 70 µm strainer before red blood cells were lysed with ammonium–chloride–potassium (ACK, Gibco, Thermo Fisher, Fairlawn, NJ, USA). Cells were washed with PBS with 1% FBS and surface receptors were stained as previously described [12,13]. Fluorescence-conjugated antibodies for CD4 and granzyme B were purchased from BioLegend (San Diego, CA, USA). Mouse IFNg ELISPOT was performed according to the manufacturer’s protocol (BD Biosciences, Franklin Lakes, NJ, USA). T cells (8 × 10^4^) were plated per well overnight in RPMI 1640 supplemented with 10% FCS. No additional stimulus was used in the elispot. As a positive control, the cells were stimulated with 5 ng/mL phorbol myristate acetate (PMA) + 1 mg/mL ionomycin (Mediatech, Inc., Manassas, VA, USA).

### 4.6. Statistical Analysis

Analysis was performed using GraphPad Prism 9.0. Treated groups were compared, with a one-way analysis, to naive mice. Differences with a *p* value of <0.05 were considered significant as determined with the Kruskal–Wallis test followed by the Dunn or Mann–Whitney test.

## Figures and Tables

**Table 1:** Effects of SV.IL-12 therapy on Pan02 metastasis.

Site	No Vector Treatment	Sindbis-IL-12
Primary tumor	++++	+
Liver	++	+
Spleen	+++	+
Perirectum	++	None
Peritoneal seeding	+	None
Lymph nodes	++	None
Periaortic	++	+
Omental	+	+
Mesenteric	++	+

Absence (none) or presence of Pan02 metastasis in various organs 3 weeks after treatments with Sindbis vectors began. Scoring is based on number of foci seen. ++++ means most of the organ is involved; +++ means significant foci; ++ or + means proportionally fewer foci. Results are based on pooled observation in groups of 10 mice for each treatment variable.

## Data Availability

The original contributions presented in this study are included in the article. Further inquiries can be directed to the corresponding author.

## Abbreviations

The following abbreviations are used in this manuscript:

SV	Sindbis Viral Vector
PDAC	Pancreatic ductal carcinoma
TME	Tumor microenvironment
TNFR	Tumor necrosis factor receptor
PanK	Pan cytokeratin
TAA	Tumor-associated antigens

## References

[1] Opp S., Hurtado A., Pampeno C., Lin Z., Meruelo D. (2022). Potent and Targeted Sindbis Virus Platform for Immunotherapy of Ovarian Cancer. Cells.

[2] Pampeno C., Opp S., Hurtado A., Meruelo D. (2024). Sindbis Virus Vaccine Platform: A Promising Oncolytic Virus-Mediated Approach for Ovarian Cancer Treatment. Int. J. Mol. Sci..

[3] Siegel R.L., Giaquinto A.N., Jemal A. (2024). Cancer statistics, 2024. CA Cancer J. Clin..

[4] Ferrone C.R., Ryan D.P. (2020). Pancreatic Cancer: A Time to Change. Ann. Surg..

[5] Guo S., Contratto M., Miller G., Leichman L., Wu J. (2017). Immunotherapy in pancreatic cancer: Unleash its potential through novel combinations. World J. Clin. Oncol..

[6] Bonaventura P., Shekarian T., Alcazer V., Valladeau-Guilemond J., Valsesia-Wittmann S., Amigorena S., Caux C., Depil S. (2019). Cold Tumors: A Therapeutic Challenge for Immunotherapy. Front. Immunol..

[7] Ebelt N.D., Zamloot V., Manuel E.R. (2020). Targeting desmoplasia in pancreatic cancer as an essential first step to effective therapy. Oncotarget.

[8] Vonderheide R.H., Bayne L.J. (2013). Inflammatory networks and immune surveillance of pancreatic carcinoma. Curr. Opin. Immunol..

[9] Ullman N.A., Burchard P.R., Dunne R.F., Linehan D.C. (2022). Immunologic Strategies in Pancreatic Cancer: Making Cold Tumors Hot. J. Clin. Oncol..

[10] Pearce E.L., Poffenberger M.C., Chang C.H., Jones R.G. (2013). Fueling immunity: Insights into metabolism and lymphocyte function. Science.

[11] Scharping N.E., Delgoffe G.M. (2016). Tumor Microenvironment Metabolism: A New Checkpoint for Anti-Tumor Immunity. Vaccines.

[12] Scherwitzl I., Hurtado A., Pierce C.M., Vogt S., Pampeno C., Meruelo D. (2018). Systemically Administered Sindbis Virus in Combination with Immune Checkpoint Blockade Induces Curative Anti-tumor Immunity. Mol. Ther. Oncolytics.

[13] Scherwitzl I., Opp S., Hurtado A.M., Pampeno C., Loomis C., Kannan K., Yu M., Meruelo D. (2020). Sindbis Virus with Anti-OX40 Overcomes the Immunosuppressive Tumor Microenvironment of Low-Immunogenic Tumors. Mol. Ther. Oncolytics.

[14] Bansal-Pakala P., Halteman B.S., Cheng M.H., Croft M. (2004). Costimulation of CD8 T cell responses by OX40. J. Immunol..

[15] Gramaglia I., Weinberg A.D., Lemon M., Croft M. (1998). Ox-40 ligand: A potent costimulatory molecule for sustaining primary CD4 T cell responses. J. Immunol..

[16] Wang Q., Shi B.M., Xie F., Fu Z.Y., Chen Y.J., An J.N., Ma Y., Liu C.P., Zhang X.K., Zhang X.G. (2016). Enhancement of CD4(+) T cell response and survival via coexpressed OX40/OX40L in Graves’ disease. Mol. Cell. Endocrinol..

[17] Zander R.A., Obeng-Adjei N., Guthmiller J.J., Kulu D.I., Li J., Ongoiba A., Traore B., Crompton P.D., Butler N.S. (2015). PD-1 Co-inhibitory and OX40 Co-stimulatory Crosstalk Regulates Helper T Cell Differentiation and Anti-Plasmodium Humoral Immunity. Cell Host Microbe.

[18] Aspeslagh S., Postel-Vinay S., Rusakiewicz S., Soria J.C., Zitvogel L., Marabelle A. (2016). Rationale for anti-OX40 cancer immunotherapy. Eur. J. Cancer.

[19] Song A., Tang X., Harms K.M., Croft M. (2005). OX40 and Bcl-xL promote the persistence of CD8 T cells to recall tumor-associated antigen. J. Immunol..

[20] Tahiliani V., Hutchinson T.E., Abboud G., Croft M., Salek-Ardakani S. (2017). OX40 Cooperates with ICOS To Amplify Follicular Th Cell Development and Germinal Center Reactions during Infection. J. Immunol..

[21] Zhang X., Xiao X., Lan P., Li J., Dou Y., Chen W., Ishii N., Chen S., Xia B., Chen K. (2018). OX40 Costimulation Inhibits Foxp3 Expression and Treg Induction via BATF3-Dependent and Independent Mechanisms. Cell Rep..

[22] Liu J., Cao S., Kim S., Chung E.Y., Homma Y., Guan X., Jimenez V., Ma X. (2005). Interleukin-12: An update on its immunological activities, signaling and regulation of gene expression. Curr. Immunol. Rev..

[23] Tseng J.C., Levin B., Hirano T., Yee H., Pampeno C., Meruelo D. (2002). In vivo antitumor activity of Sindbis viral vectors. J. Natl. Cancer Inst..

[24] Tseng J.C., Levin B., Hurtado A., Yee H., Perez de Castro I., Jimenez M., Shamamian P., Jin R., Novick R.P., Pellicer A. (2004). Systemic tumor targeting and killing by Sindbis viral vectors. Nat. Biotechnol..

[25] Venticinque L., Meruelo D. (2010). Sindbis viral vector induced apoptosis requires translational inhibition and signaling through Mcl-1 and Bak. Mol. Cancer.

[26] Hurtado A., Tseng J.C., Meruelo D. (2006). Gene therapy that safely targets and kills tumor cells throughout the body. Rejuvenation Res..

[27] Tseng J.C., Hurtado A., Yee H., Levin B., Boivin C., Benet M., Blank S.V., Pellicer A., Meruelo D. (2004). Using sindbis viral vectors for specific detection and suppression of advanced ovarian cancer in animal models. Cancer Res..

[28] Lundstrom K. (2022). Alphaviruses in Cancer Therapy. Front. Mol. Biosci..

[29] Lundstrom K. (2022). Alphaviruses in Immunotherapy and Anticancer Therapy. Biomedicines.

[30] Pampeno C., Hurtado A., Opp S., Meruelo D. (2023). Channeling the Natural Properties of Sindbis Alphavirus for Targeted Tumor Therapy. Int. J. Mol. Sci..

[31] Jose J., Snyder J.E., Kuhn R.J. (2009). A structural and functional perspective of alphavirus replication and assembly. Future Microbiol..

[32] Strauss J.H., Strauss E.G. (1994). The alphaviruses: Gene expression, replication, and evolution. Microbiol. Rev..

[33] Zimmerman O., Holmes A.C., Kafai N.M., Adams L.J., Diamond M.S. (2023). Entry receptors—The gateway to alphavirus infection. J. Clin. Invest..

[34] Tomioka D., Maehara N., Kuba K., Mizumoto K., Tanaka M., Matsumoto K., Nakamura T. (2001). Inhibition of growth, invasion, and metastasis of human pancreatic carcinoma cells by NK4 in an orthotopic mouse model. Cancer Res..

[35] Corbett T.H., Roberts B.J., Leopold W.R., Peckham J.C., Wilkoff L.J., Griswold D.P., Schabel F.M. (1984). Induction and chemotherapeutic response of two transplantable ductal adenocarcinomas of the pancreas in C57BL/6 mice. Cancer Res..

[36] Greco S.H., Tomkötter L., Vahle A.K., Rokosh R., Avanzi A., Mahmood S.K., Deutsch M., Alothman S., Alqunaibit D., Ochi A. (2015). TGF-β Blockade Reduces Mortality and Metabolic Changes in a Validated Murine Model of Pancreatic Cancer Cachexia. PLoS ONE.

[37] Pham T.N.D., Shields M.A., Spaulding C., Principe D.R., Li B., Underwood P.W., Trevino J.G., Bentrem D.J., Munshi H.G. (2021). Preclinical Models of Pancreatic Ductal Adenocarcinoma and Their Utility in Immunotherapy Studies. Cancers.

[38] Suklabaidya S., Dash P., Das B., Suresh V., Sasmal P.K., Senapati S. (2018). Experimental models of pancreatic cancer desmoplasia. Lab. Invest..

[39] Torres M.P., Rachagani S., Souchek J.J., Mallya K., Johansson S.L., Batra S.K. (2013). Novel pancreatic cancer cell lines derived from genetically engineered mouse models of spontaneous pancreatic adenocarcinoma: Applications in diagnosis and therapy. PLoS ONE.

[40] Yu M., Scherwitzl I., Opp S., Tsirigos A., Meruelo D. (2019). Molecular and metabolic pathways mediating curative treatment of a non-Hodgkin B cell lymphoma by Sindbis viral vectors and anti-4-1BB monoclonal antibody. J. Immunother. Cancer.

[41] Ishihara J., Ishihara A., Potin L., Hosseinchi P., Fukunaga K., Damo M., Gajewski T.F., Swartz M.A., Hubbell J.A. (2018). Improving Efficacy and Safety of Agonistic Anti-CD40 Antibody Through Extracellular Matrix Affinity. Mol. Cancer Ther..

[42] O’Hara M.H., O’Reilly E.M., Varadhachary G., Wolff R.A., Wainberg Z.A., Ko A.H., Fisher G., Rahma O., Lyman J.P., Cabanski C.R. (2021). CD40 agonistic monoclonal antibody APX005M (sotigalimab) and chemotherapy, with or without nivolumab, for the treatment of metastatic pancreatic adenocarcinoma: An open-label, multicentre, phase 1b study. Lancet Oncol..

[43] Thoidingjam S., Bhatnagar A.R., Sriramulu S., Siddiqui F., Nyati S. (2024). Optimizing Pancreatic Cancer Therapy: The Promise of Immune Stimulatory Oncolytic Viruses. Int. J. Mol. Sci..

[44] Zhang Y., Li Y., Chen K., Qian L., Wang P. (2022). Oncolytic virotherapy against the tumor microenvironment and its potential in pancreatic cancer. J. Cancer Res. Ther..

[45] Bhatnagar A.R., Siddiqui F., Khan G., Pompa R., Kwon D., Nyati S. (2024). Long-Term Follow-Up of Phase I Trial of Oncolytic Adenovirus-Mediated Cytotoxic and Interleukin-12 Gene Therapy for Treatment of Metastatic Pancreatic Cancer. Biomedicines.

[46] Musher B.L., Rowinsky E.K., Smaglo B.G., Abidi W., Othman M., Patel K., Jawaid S., Jing J., Brisco A., Leen A.M. (2024). LOAd703, an oncolytic virus-based immunostimulatory gene therapy, combined with chemotherapy for unresectable or metastatic pancreatic cancer (LOKON001): Results from arm 1 of a non-randomised, single-centre, phase 1/2 study. Lancet Oncol..

[47] Liu S., Li F., Ma Q., Du M., Wang H., Zhu Y., Deng L., Gao W., Wang C., Liu Y. (2023). OX40L-Armed Oncolytic Virus Boosts T-cell Response and Remodels Tumor Microenvironment for Pancreatic Cancer Treatment. Theranostics.

[48] Ma Y., Li J., Wang H., Chiu Y., Kingsley C.V., Fry D., Delaney S.N., Wei S.C., Zhang J., Maitra A. (2020). Combination of PD-1 Inhibitor and OX40 Agonist Induces Tumor Rejection and Immune Memory in Mouse Models of Pancreatic Cancer. Gastroenterology.

[49] Choi Y., Chang J. (2013). Viral vectors for vaccine applications. Clin. Exp. Vaccine Res..

[50] Gardner J.P., Frolov I., Perri S., Ji Y., MacKichan M.L., zur Megede J., Chen M., Belli B.A., Driver D.A., Sherrill S. (2000). Infection of human dendritic cells by a sindbis virus replicon vector is determined by a single amino acid substitution in the E2 glycoprotein. J. Virol..

[51] Granot T., Yamanashi Y., Meruelo D. (2014). Sindbis viral vectors transiently deliver tumor-associated antigens to lymph nodes and elicit diversified antitumor CD8+ T-cell immunity. Mol. Ther..

[52] MacDonald G.H., Johnston R.E. (2000). Role of dendritic cell targeting in Venezuelan equine encephalitis virus pathogenesis. J. Virol..

[53] Osada T., Morse M.A., Hobeika A., Lyerly H.K. (2012). Novel recombinant alphaviral and adenoviral vectors for cancer immunotherapy. Semin. Oncol..

[54] Pushko P., Parker M., Ludwig G.V., Davis N.L., Johnston R.E., Smith J.F. (1997). Replicon-helper systems from attenuated Venezuelan equine encephalitis virus: Expression of heterologous genes in vitro and immunization against heterologous pathogens in vivo. Virology.

[55] Uematsu Y., Vajdy M., Lian Y., Perri S., Greer C.E., Legg H.S., Galli G., Saletti G., Otten G.R., Rappuoli R. (2012). Lack of interference with immunogenicity of a chimeric alphavirus replicon particle-based influenza vaccine by preexisting antivector immunity. Clin. Vaccine Immunol..

[56] Hardwick J.M., Levine B. (2000). Sindbis virus vector system for functional analysis of apoptosis regulators. Methods Enzymol..

[57] Sane J., Kurkela S., Lokki M.L., Miettinen A., Helve T., Vaheri A., Vapalahti O. (2012). Clinical Sindbis alphavirus infection is associated with HLA-DRB1*01 allele and production of autoantibodies. Clin. Infect. Dis..

[58] Strauss J.H., Wang K.S., Schmaljohn A.L., Kuhn R.J., Strauss E.G. (1994). Host-cell receptors for Sindbis virus. Arch. Virol. Suppl..

[59] Bredenbeek P.J., Frolov I., Rice C.M., Schlesinger S. (1993). Sindbis virus expression vectors: Packaging of RNA replicons by using defective helper RNAs. J. Virol..

[60] Frolov I., Hoffman T.A., Prágai B.M., Dryga S.A., Huang H.V., Schlesinger S., Rice C.M. (1996). Alphavirus-based expression vectors: Strategies and applications. Proc. Natl. Acad. Sci. USA.

[61] Hurtado A., Tseng J.C., Boivin C., Levin B., Yee H., Pampeno C., Meruelo D. (2005). Identification of amino acids of Sindbis virus E2 protein involved in targeting tumor metastases in vivo. Mol. Ther..

